# 

**Published:** 1858-10

**Authors:** 


### PROFESSIONAL APPOINTMENTS.

The chair of surgery in the National Medical College, at Washington, made vacant by the resignation of Prof. May, has been filled by the appointment of Dr. John G. F. Holston. The chair of chemistry in the Virginia Medical College has been filled by the appointment of Dr. James B. McCaw.

